# Efgartigimod-alfa therapy in acetylcholine receptor antibody myasthenia gravis: Romanian experience

**DOI:** 10.3389/fneur.2026.1945691

**Published:** 2026-09-03

**Authors:** Nicolae Grecu, Oana Antonia Mihalache, Eugenia Irene Davidescu, Rodica Bălașa, Mihaela Simu, Vitalie Văcăraș, Ileana-Maria Vodă, Andreea-Ioana Mușuroi, Andra Mădălina Ene, Diana Mihaela Petrescu, Cristina Elena Mitu, Lorena Furnică, Amalia Cornea, Romana Homorodean, Crisanda Vîlciu, Cristina Tiu

**Affiliations:** 1 Department of Neurology, University Emergency Hospital Bucharest, Bucharest, Romania; 2 Department of Neurology, Fundeni Clinical Institute, Bucharest, Romania; 3 Department of Clinical Neurosciences, Carol Davila University of Medicine and Pharmacy, Bucharest, Romania; 4 Department of Neurology, Colentina Clinical Hospital, Bucharest, Romania; 5 George Emil Palade University of Medicine, Pharmacy, Science and Technology, Târgu Mureș, Romania; 6 First Clinic of Neurology, Mureș County Emergency Clinical Hospital, Târgu Mureș, Romania; 7 Victor Babeș University of Medicine and Pharmacy, Timișoara, Romania; 8 Pius Brînzeu Timișoara County Emergency Clinical Hospital, Timișoara, Romania; 9 NeuroTim Med, Timișoara, Romania; 10 Department of Neurosciences, Iuliu Hațieganu University of Medicine and Pharmacy, Cluj-Napoca, Romania; 11 Department of Neurology, County Emergency Clinical Hospital, Cluj-Napoca, Romania

**Keywords:** corticosteroid sparing, efgartigimod, FcRn inhibitor, myasthenia gravis, real-world data

## Abstract

**Background:**

Efgartigimod alfa, the first neonatal Fc receptor inhibitor approved for the treatment of acetylcholine receptor antibody (AChR)-positive generalized myasthenia gravis (MG), has demonstrated efficacy in randomized controlled trials. However, real-world data, particularly from Eastern Europe, remain limited.

**Methods:**

This prospective, multicenter study enrolled AChR-positive generalized MG patients treated with efgartigimod across six Romanian university hospitals between March 2024 and September 2025. Outcomes were assessed using the Myasthenia Gravis Activities of Daily Living (MG-ADL) scale, the Quantitative Myasthenia Gravis (QMG) score, and the Myasthenia Gravis Quality of Life 15-item revised scale (MG-QoL15r). Clinically meaningful improvement (CMI), minimal symptom expression (MSE), patient-acceptable symptom state (PASS), subdomain response, and corticosteroid sparing were evaluated.

**Results:**

Thirty-nine patients completed at least one treatment cycle, receiving a total of 210 cycles. In cycle 1, CMI rates were 89.7% for MG-ADL and 74.4% for QMG, with a median MG-ADL reduction of 4.0 points (*p* < 0.001); 69.2% achieved CMI after a single infusion. Composite PASS increased from 25.6% in cycle 1 to 54.5% in cycle 8. Subdomain analysis showed improvements across all domains for both the MG-ADL and the QMG, with the exception of QMG respiratory. Among patients on corticosteroids (*n* = 29), median prednisone dose decreased from 20.0 to 10.0 mg/day (*p* = 0.004), with 58.6% achieving reduction or discontinuation.

**Conclusion:**

This first Eastern European real-world efgartigimod-treated cohort confirms rapid and sustained improvement and corticosteroid sparing, consistent with prior evidence.

## Introduction

Myasthenia gravis (MG) is a rare, chronic autoimmune disease and represents the most prevalent disorder affecting the neuromuscular junction (1–3). In approximately 80–85% of patients with generalized disease pathogenic autoantibodies against the acetylcholine receptor (AChR) are detected (2, 4, 5). These antibodies impair neuromuscular transmission by inducing complement-mediated postsynaptic membrane damage and antibody-induced AChR cross-linking, leading to subsequent internalization and degradation, ultimately reducing the density of functional receptors at the neuromuscular junction (6–8).

The primary goal of treatment is to achieve satisfactory symptom control while restoring quality of life and minimizing the risk of adverse events (9, 10). Conventional therapy includes acetylcholinesterase inhibitors (AChEIs) and immunosuppressive therapy (corticosteroids and steroid-sparing immunosuppressants) (9, 11, 12), but up to 15% are considered therapy-refractory (13, 14). The limited efficacy and adverse effects of conventional therapies have led to the development of targeted treatment strategies, including B-cell-directed therapies, complement inhibitors, and neonatal Fc receptor (FcRn) antagonists. FcRn inhibitors reduce pathogenic IgG levels by disrupting the normal FcRn-mediated IgG recycling pathway, which, under physiological conditions, rescues IgG from lysosomal degradation and returns it to the circulation (15, 16).

Efgartigimod, the first FcRn inhibitor to be approved for the treatment of MG, has shown significant improvement over placebo from a clinical and conventional therapy reduction standpoint in AChR-MG in the pivotal phase III ADAPT trial (17, 18). Its open-label extension and subsequent publications of real-world data have confirmed the added benefit of this drug in the management of MG.

This was a prospective multicenter study undertaken in six tertiary centers in Romania in order to assess the effect of efgartigimod in AChR-positive MG patients. The primary objective was to evaluate the effectiveness and safety of efgartigimod alfa in routine clinical practice and to document treatment-emergent adverse events outside randomized controlled trials. Access to efgartigimod alfa was provided through a donation-based program, enabling the assessment of real-world therapeutic effectiveness and tolerability.

## Materials and methods

This was a multicentric study that was undertaken in six university hospitals in Romania. Inclusion criteria included: age ≥18 years; AChR-positive, MGFA II to IV MG, with an MG-ADL score of ≥5 (with ≥50% from non-ocular symptoms or ≥2 non-ocular items with ≥2 points); AChEI therapy and at least one failed immunosuppressant therapy (defined as lack of efficacy or adverse effects). Exclusion criteria included: the presence of MuSK or LRP4 antibodies; pregnancy; hepatitis B, hepatitis C or HIV infections; monoclonal antibody therapy within the 6 months prior to enrollment; IVIG or PLEX within the month prior to enrollment.

Participants were assessed through clinical examination at the first and last infusion of each cycle, routine blood and urine analysis at first infusion, MG-ADL and MG-QoL15r at all infusions, and QMG and MMT at the first and last infusion. They were subsequently administered the MG-ADL at week 7 after the first infusion of the previous cycle and, if they met the retreatment criteria, the protocol was restarted for the next treatment cycle.

After the first cycle, participants were given another round of therapy if their MG-ADL score was at least 5 points or if there was an at least 2 point increase in the same score as compared to their previous cycle end score. In select cases, retreatment was left at the treating physician’s discretion even if clinical scores did not meet retreatment threshold.

Demographic, medical (pertaining to the MG and also to comorbidities), treatment efficacy (clinical scores), and safety data were recorded. Background MG medication changes were left at the discretion of the treating neurologists, and AChEI, azathioprine, and mycophenolate mofetil doses were recorded at the start and end of each treatment cycle.

Efgartigimod was provided as a donation by the producer after written request from the participating hospitals; intravenous formulation was administered to all participants.

Data analysis was performed in R (version 4.5.2, R Foundation for Statistical Computing) through R Studio (Posit Software, version 2026.01.1). Continuous variables were tested for normality using the Shapiro–Wilk test. Normally distributed variables are reported as mean ± SD; non-normally distributed variables as median [Q1–Q3]. Categorical variables are reported as *n* (%).

A complete treatment cycle was defined as four weekly infusions of efgartigimod. Incomplete cycles (fewer than four infusions) were excluded from the per-cycle efficacy analysis. Cycle numbering was based on completed cycles only. Inter-cycle intervals were calculated between the first infusion of consecutive cycles.

Clinically meaningful improvement (CMI) was defined as a ≥ 2 point reduction in MG-ADL or a ≥ 3 point reduction in QMG from the first to the last infusion of the respective cycle. Response rates are reported with 95% Wilson confidence intervals. Minimal symptom expression (MSE) was defined as MG-ADL ≤ 1 point, and patient-acceptable symptom state (PASS) was defined as MG-ADL ≤ 2 points, QMG ≤ 8 points, and MG-QoL15r ≤ 6 points, as previously described (19, 20).

Speed of response in the first cycle of treatment was assessed by comparing MG-ADL scores at each visit to baseline score using the Wilcoxon signed-rank test. For longitudinal analysis of response, paired comparisons of scores between cycle start and cycle end were also performed using the Wilcoxon signed-rank test due to the non-normal distribution of the scores. MG-ADL and QMG items were grouped for subdomain analysis as per Bril et al. (21). Corticosteroid sparing was assessed by comparing prednisone dose at enrollment and at the end of the last treatment cycle.

*Post-hoc* exploratory analyses assessed treatment response by age of MG onset and thymus histology. Differences between groups were tested using the chi-square test for CMI rates and the Kruskal-Wallis or Mann–Whitney U test for continuous variables.

Baseline demographic and clinical characteristics were compared with published efgartigimod cohorts using summary-level statistics extracted from original full-text articles. These were exploratory and intended to contextualize results and not to establish causal relationships.

All statistical tests were two-sided with a significance level of *α* = 0.05.

## Results

### Patient data

Forty one patients were included between March and December 2024, and followed-up for up to 18 months from the first enrollment (up to September 2025). Baseline demographic data are presented in Table 1. Mean age at inclusion was 51.1 ± 17.0 years, with the majority (61.0%) of participants being females. Most patients had MGFA class II disease (58.5%) with a median MG-ADL of 7.0 [6.0–10.0]. Bulbar symptoms were the most common presentation at disease onset (39.0%) and thymoma-associated MG accounted for 12.2% of cases. 53.7% of participants had a history of myasthenic crisis, and 73.2% were receiving corticosteroids at the time of inclusion.

**Table 1:** Demographic data for the patient cohort.

Parameter	Value	
Age at inclusion (years)	51.1 ± 17.0 (range 18.1–77.5)	
Gender	Female Male	25 (61.0%) 16 (39.0%)
Place of residence	Urban Rural	28 (68.3%) 13 (31.7%)
Disease duration prior to inclusion (years)	6.0 [3.0–15.0] (range 0.0–40.0)	
Onset-to-diagnosis delay (years)	0.0 [0.0–1.0] (range 0.0–6.0)	
Symptoms at onset	Ocular Bulbar Limb Generalized	11 (26.8%) 16 (39.0%) 7 (17.1%) 7 (17.1%)
MGFA class at inclusion	IIA IIB IIIA IIIB IVB	5 (12.2%) 19 (46.3%) 9 (22.0%) 6 (14.6%) 2 (4.9%)
MG-ADL at inclusion	7.0 [6.0–10.0] (range 5.0–13.0)	
Thymus status at inclusion*	Absent Remaining Thymectomy No data	8 (19.5%) 12 (29.3%) 19 (46.3%) 2 (4.9%)
Histology after thymectomy	Normal Hyperplasia Thymoma No data	2 (10.5%) 6 (31.6%) 5 (26.3%) 6 (31.6%)
Treatment at inclusion	AChEI AChEI dose† CS CS dose†‡ NCSIS IVIG/PLEX	41 (100%) 240.0 [180.0–300.0] (range 150.0–720.0) 30 (73.2%) 20.0 [10.0–30.0] (range 5.0–40.0) 14 (34.1%) 12 (29.3%)
Prior immunosuppressant exposure	13 (31.7%)	
History of myasthenic crisis	22 (53.7%)	
Number of crises‡	2.0 [2.0–3.0] (range 1.0–7.0)	
Treatment-related AEs	31 (75.6%)	
Autoimmune comorbidities	7 (17.1%)	

Continuous variables: mean ± SD if normally distributed, median [IQR] if non-normally distributed.

*As assessed through thorax CT scan; †Dose in mg/day (prednisone equivalent for CS); ‡Corticosteroid dose reported for users only (*n* = 30) and number of crises reported for patients with history of myasthenic crisis only (*n* = 22).

AChEI, acetylcholinesterase inhibitor; AE, adverse event; AZA, azathioprine; CS, corticosteroid; NCSIS, non-corticosteroid immunosuppressant; MG-ADL, Myasthenia Gravis Activities of Daily Living; MGFA, Myasthenia Gravis Foundation of America; MMF, mycophenolate mofetil.

### Treatment cycle data

**Figure 1:**
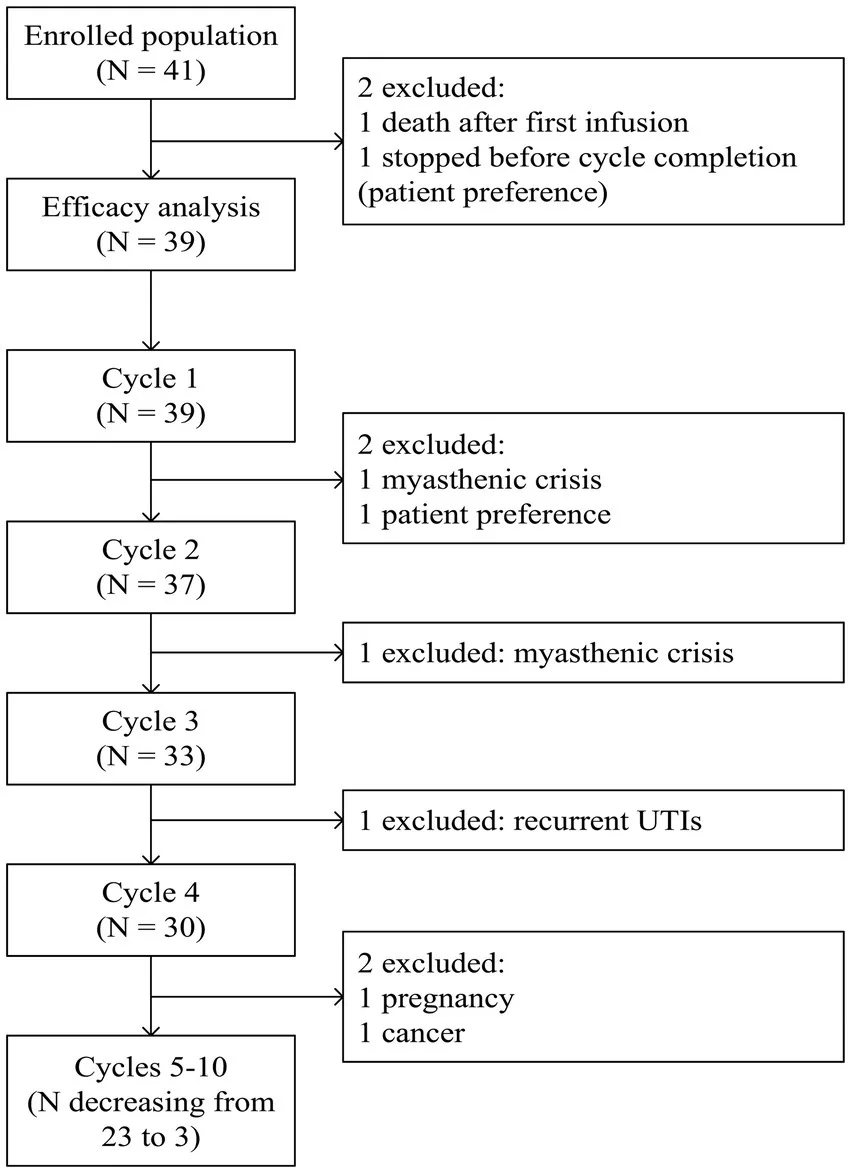
Study flowchart. Unless otherwise specified, the decrease in patient numbers between cycles was due to retreatment criteria not being met (MG-ADL score <5 and <2-point increase from previous cycle end).

Two patients were excluded from the final efficacy analysis as they did not finish a complete treatment cycle; these and further exclusions and cycle interruptions are shown in Figure 1. A total of 210 complete cycles were administered, with a median of 5.0 [IQR 4.0–8.0] (range 1–10) cycles per patient; 13 (7.6%) of the 171 retreatment cycles were administered at physician discretion. Median inter-cycle interval (between the first infusion of consecutive cycles) was 56 [IQR 49–66] (range 47–225) days.

### Treatment response

**Figure 2:**
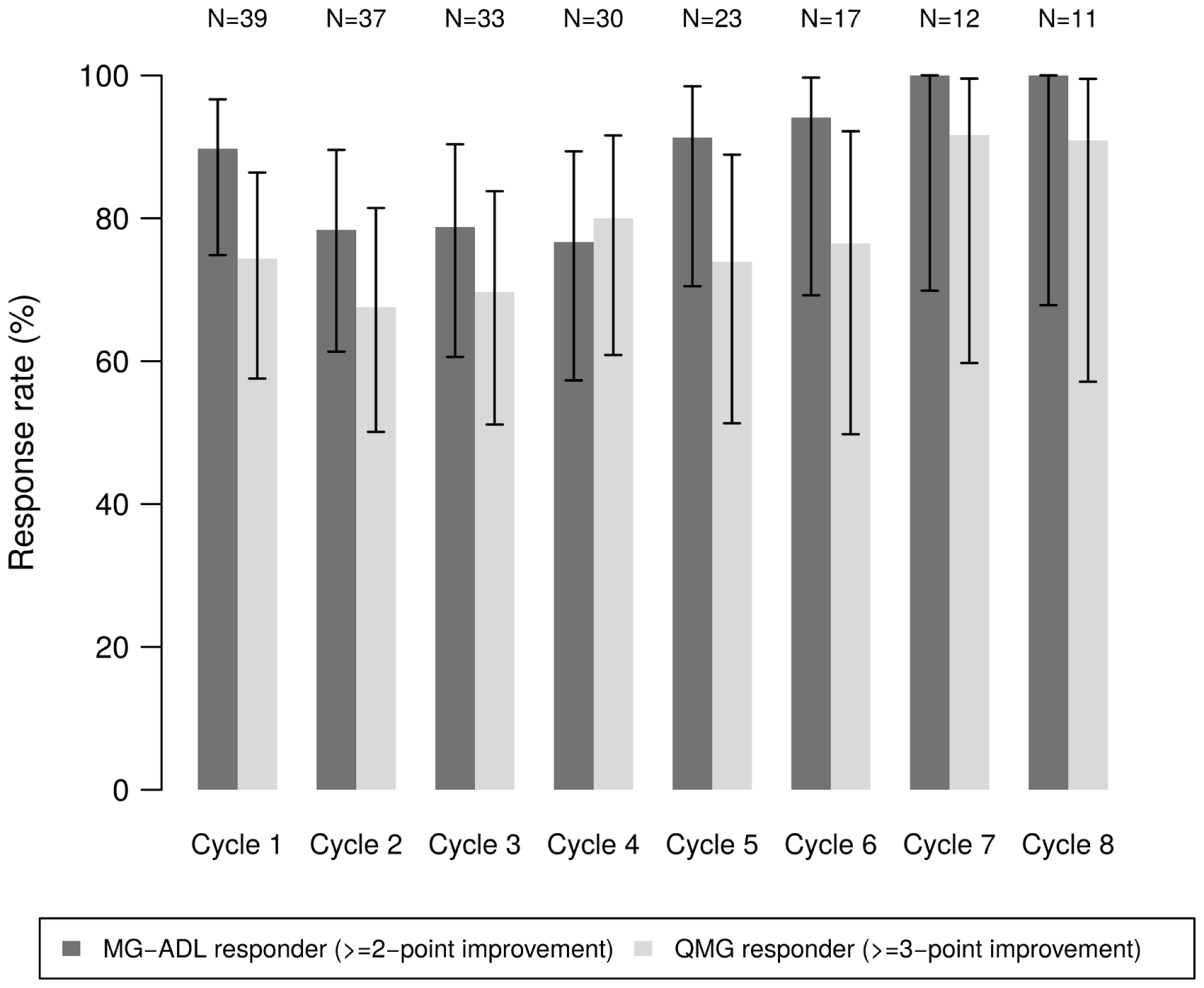
Percentage of responders for cycles 1–8.

**Figure 3:**
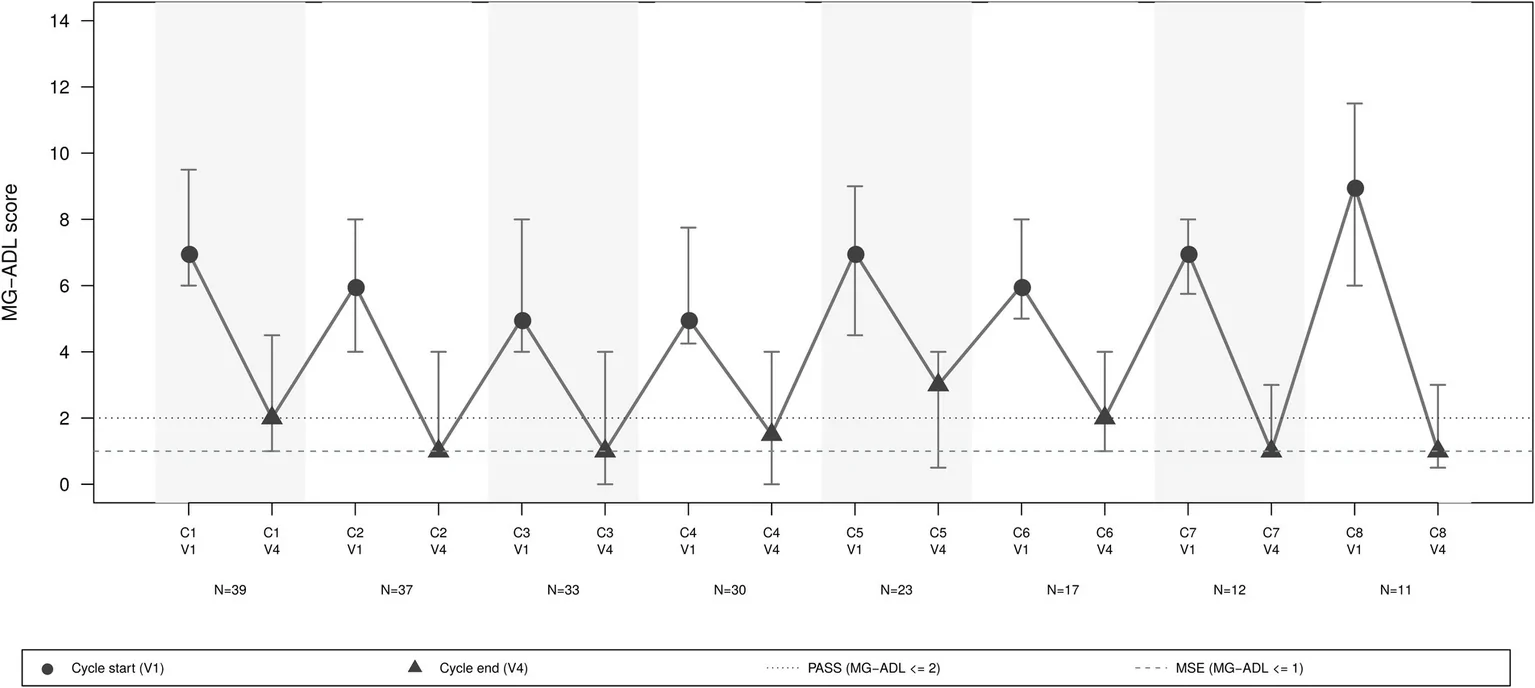
Longitudinal evolution of median MG-ADL scores across treatment cycles 1–8. Error bars represent interquartile range (Q1 - Q3). *N* = number of patients per cycle.

MG-ADL and QMG response rates across treatment cycles are presented in Figure 2; complete score evolutions are presented in Table 2. For the first cycle, CMI thresholds were reached by 89.7% (95% CI 74.8–96.7%) for MG-ADL and 74.4% (95% CI 57.6–86.4%) patients for QMG, respectively. Response rates remained consistent across subsequent cycles, with MG-ADL response exceeding 76% and QMG response exceeding 67% throughout cycles 2–8. The longitudinal evolution of MG-ADL scores along treatment cycles is shown in Figure 3.

**Table 2:** Median score evolution from cycle start (1st infusion) to cycle end (4th infusion).

Cycle no. (participants)	MG-ADL				QMG				MG-QoL15r				MMT			
	1st infusion	4th infusion	Change	*p*-value	1st infusion	4th infusion	Change	*p-*value	1st infusion	4th infusion	Change	*p*-value	1st infusion	4th infusion	Change	*p*-value
Cycle 1 (*n* = 39)	7.0	2.0	4.0	<0.001	14.0	8.0	6.0	<0.001	20.0	7.0	9.0	<0.001	22.0	8.0	12.0	<0.001
Cycle 2 (*n* = 37)	6.0	1.0	3.0	<0.001	12.0	7.0	5.0	<0.001	12.0	6.0	4.0	<0.001	15.0	8.0	5.0	<0.001
Cycle 3 (*n* = 33)	5.0	1.0	3.0	<0.001	12.0	7.0	5.0	<0.001	10.0	5.0	3.0	<0.001	17.0	8.0	6.0	<0.001
Cycle 4 (*n* = 30)	5.0	1.5	3.0	<0.001	11.5	6.0	6.0	<0.001	11.0	6.0	4.0	<0.001	16.0	6.5	6.0	<0.001
Cycle 5 (*n* = 23)	7.0	3.0	4.0	<0.001	14.0	7.0	5.0	<0.001	11.0	7.0	4.0	<0.001	16.0	8.0	6.0	<0.001
Cycle 6 (*n* = 17)	6.0	2.0	4.0	<0.001	13.0	9.0	4.0	0.003	15.0	6.0	6.0	<0.001	14.0	6.0	4.0	0.010
Cycle 7 (*n* = 12)	7.0	1.0	5.0	0.002	14.0	7.0	7.5	0.002	12.0	6.0	6.5	0.003	14.5	4.0	9.5	0.003
Cycle 8 (*n* = 11)	9.0	1.0	7.0	0.004	13.0	6.0	7.0	0.004	14.0	4.0	11.0	0.006	14.0	6.0	12.0	0.006
Cycle 9 (*n* = 5)	5.5	1.0	4.5	0.100	12.5	4.5	8.0	0.100	12.5	3.5	6.5	0.100	8.5	0.0	8.5	0.100
Cycle 10 (*n* = 3)	9.0	2.0	6.0	0.181	11.0	5.0	8.0	0.414	17.0	11.0	4.0	0.371	13.0	7.0	10.0	0.181

Values represent medians. Change = median of individual patient differences (V1 − V4). Wilcoxon signed-rank test. Cycles 9–10 did not reach statistical significance due to small sample size.

When assessing first cycle response speed, 69.2% of participants had a ≥ 2-point reduction for MG-ADL after the first infusion, and this percentage increased to 87.2% after the second infusion and 89.7% after the third infusion.

In cycle 1, the median MG-ADL score decreased from 7.0 [IQR 6.0–9.5] to 2.0 [IQR 1.0–4.5], with a median change of 4.0 points (*p* < 0.001), while the median QMG score decreased from 14.0 [IQR 11.0–17.5] to 8.0 [IQR 4.5–10.0], with a median change of 6.0 points (*p* < 0.001). Beyond the CMI threshold, 76.9% of patients achieved a ≥ 3-point MG-ADL reduction and 56.4% a ≥ 5-point QMG reduction (Supplementary material 1).

MSE was achieved by 33.3–58.3% of participants depending on treatment cycle. For PASS, individual score thresholds were achieved by 47.8–80% for MG-ADL, 47.1–80.0% for QMG, and 0–81.8% for MG-QoL15r, with MG-QOL15r scores consistently being lower that the others; composite PASS thresholds increased from 25.6% at the end of cycle 1 to 54.5% at the end of cycle 8 (Table 3).

**Table 3:** MSE and PASS achievement.

Cycle no. (participants)	MSE	PASS			
		MG-ADL criterion	QMG criterion	MG-QoL15r criterion	combined
Cycle 1 (*n* = 39)	38.5%	56.4%	53.8%	43.6%	25.6%
Cycle 2 (*n* = 37)	54.1%	62.2%	64.9%	51.4%	35.1%
Cycle 3 (*n* = 33)	51.5%	63.6%	63.6%	51.5%	39.4%
Cycle 4 (*n* = 30)	50.0%	63.3%	70.0%	56.7%	43.3%
Cycle 5 (*n* = 23)	34.8%	47.8%	56.5%	43.5%	26.1%
Cycle 6 (*n* = 17)	35.3%	52.9%	47.1%	52.9%	29.4%
Cycle 7 (*n* = 12)	58.3%	66.7%	58.3%	50.0%	33.3%
Cycle 8 (*n* = 11)	54.5%	72.7%	63.6%	81.8%	54.5%
Cycle 9 (*n* = 5)	40.0%	80.0%	80.0%	60.0%	60.0%
Cycle 10 (*n* = 3)	33.3%	66.7%	66.7%	0.0%	0.0%

MSE, minimal symptom expression (defined as MG-ADL ≤ 1 points); PASS, patient acceptable symptom state (defined as MG-ADL ≤ 2 points, QMG ≤ 8 points, and/or MG-QoL15r ≤ 6 points).

Subdomain response analysis was performed for cycles 1–8 (Supplementary material 2); cycles 9 and 10 were excluded due to the small number of patients. For MG-ADL, significant improvements were seen in all subdomains for all treatment cycles, with the bulbar subdomain showing the largest and most consistent change (median change 1.0–2.5 per cycle, *p* < 0.01 for all cycles). For QMG, improvements were seen for all domains with the exception of the respiratory subdomain, in which changes did not reach statistical significance in any cycle (*p* > 0.05); however, when examining only participants with a ≥ 1 respiratory domain QMG score a significant reduction was seen for cycle 1 (*p* = 0.016, *n* = 8).

Post-hoc exploratory analysis assessing treatment response by age of MG onset and thymus histology showed similar CMI rates in the early-onset (EOMG) and late-onset (LOMG) subgroups (95.8 and 100%, respectively), but lower in the very late-onset (VLOMG) subgroup (50.0%; *p* = 0.002 across groups); however, the number of VLOMG participants was small (*n* = 6). CMI did not differ by thymectomy status (94.7% vs. 85.0%, *p* = 0.605) (Supplementary material 3).

### Symptomatic and immunosuppressant treatment changes

**Figure 4:**
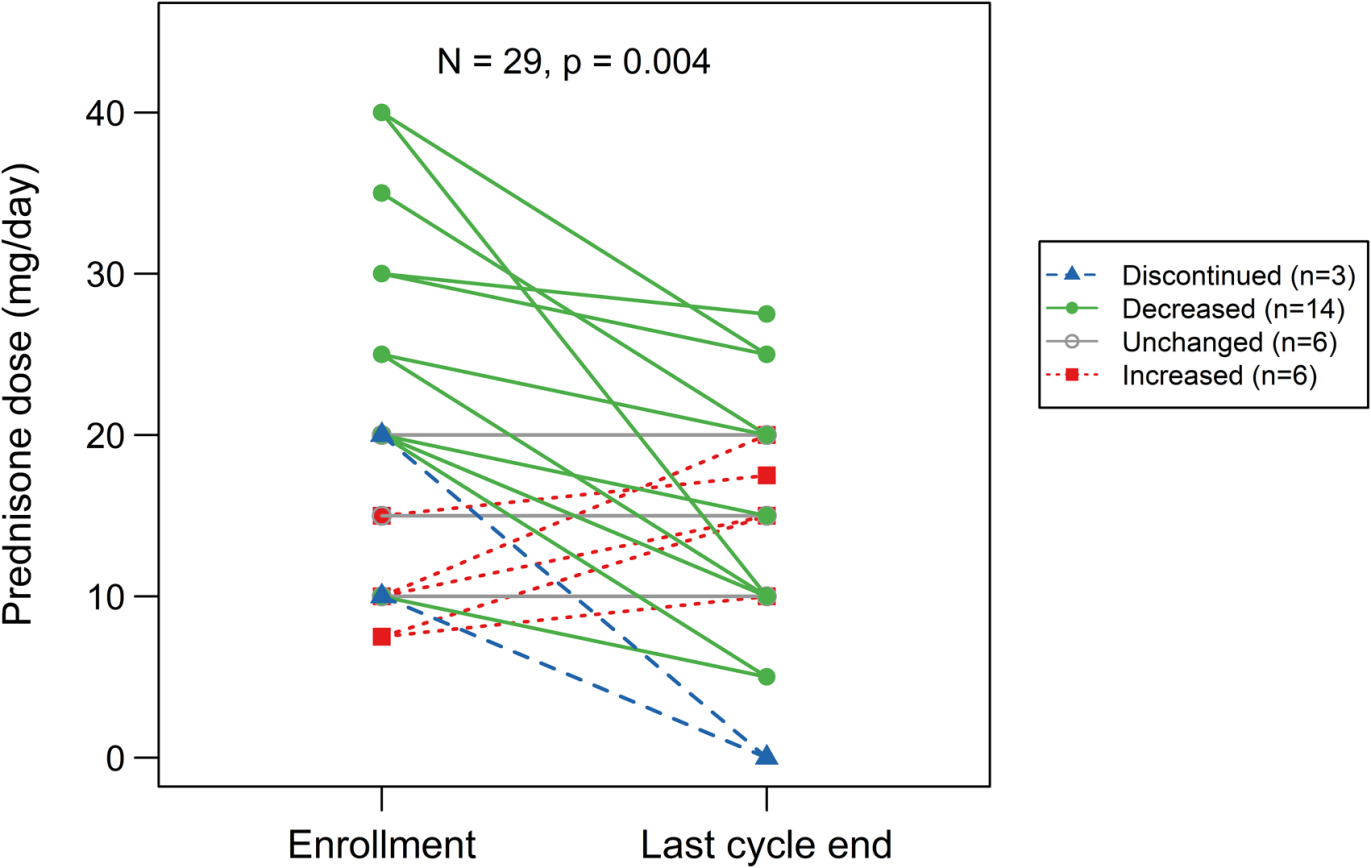
Individual Prednisone dose changes from enrollment to end of last treatment cycle. Each line represents one patient.

There were 30 patients on corticosteroids at enrollment; however, only 29 were included in the analysis, as one was among those who did not complete a full treatment cycle. Median prednisone dose decreased from 20.0 mg/day [IQR 10.0–25.0] at start of therapy to 10.0 mg/day [IQR 10.0–17.5] at the end of the last treatment cycle (*p* = 0.004), with an overall median change of −5.0 mg/day. 17 (58.6%) patients achieved corticosteroid reduction or discontinuation, with three of those stopping treatment altogether (Figure 4).

AChEI doses remained unchanged in 74.4% of patients, decreased in 20.5%, and increased in 5.1%. NCSIS doses remained stable in 81.8% (9/11) patients on azathioprine and 66.7% (2/3) patients on mycophenolate mofetil. While the overall AChEI dose reduction was statistically significant (*p* = 0.026), improvement in MG-ADL was similar in patients with and without AChEI dose changes (median 5.5 vs. 5.0 points, *p* = 0.340); the small number of patients with NCSIS changes limits interpretation (Supplementary material 4).

### Cycle interruptions and safety analysis

There were two deaths in the cohort. The first occurred two days after the first infusion of the first cycle of therapy, was ruled as sudden cardiac death and was ascribed to significant cardiovascular comorbidities. The second was due to myasthenic crisis and secondary ventilator-associated pneumonia which occurred after the first infusion of the third cycle of therapy. Both deaths are unlikely to be related to the administration of the drug as per the WHO-UMC criteria, as they can be ascribed to comorbidities in the first case, and to MG in the second (Table 2).

As shown in Figure 1, treatment was discontinued due to patient preference in two participants (mainly citing difficulty in travelling to the weekly infusions), myasthenic crisis (two, one after the first cycle, and one after the second cycle), pregnancy (one), and newly-diagnosed breast cancer. One patient had recurrent urinary tract infections, with one instance of sepsis; when assessing causality, this is possibly related to the use of efgartigimod, as susceptibility to infections is a known side effect of the drug. There were four additional cycle interruptions: one, after hepatic enzymes elevation during the first cycle of therapy; three due to respiratory tract infections.

Two patients presented adverse events (one arterial hypotension, one headache) during several infusion, but did not require treatment discontinuation.

## Discussion

In our group a ≥ 2 point MG-ADL was achieved by 89.7% of patients, while previous reports show CMI rates in 67.7–98.7% of participants (21–27). The degree of MG-ADL and QMG reduction was comparable to those reported by Sgarzi et al. (28), Katyal et al. (23), and Pane et al. (29). While cohorts were different from several standpoints, variability in reported response rates is probably primarily driven by differences in response definitions, assessment timing, and refractory status, rather than by any single demographic or disease characteristic, as cross-study Spearman correlations showed no significant association between CMI rates and age, gender, MGFA class, baseline MG-ADL, thymectomy rate, or thymoma (Supplementary material 5).

MSE achievement rates in the first cycle approached those reported in the ADAPT trial, and were greater than that reported in other studies (23). To our knowledge, the PASS has not been previously assessed in the setting of treatment with efgartigimod. As expected, the percentage of participants fulfilling the MG-ADL threshold were higher than for MSE, but the fact that MG-QoL15r achievement rates were consistently lower than for the two other scales was interesting and likely reflects the broader quality of life impairment.

Subdomain analysis results were consistent with those of the post-hoc analyses of the ADAPT trial, with reduction of scores on all MG-ADL and QMG divisions, maximal benefit on MG-ADL bulbar scores, and non-statistically significant improvements in the QMG respiratory score. While there was a statistically significant improvement when considering only participants with a ≥ 1 respiratory QMG score, their number was low and this precludes a firm conclusion. Furthermore, the inherent limitations of the forced vital capacity as sole parameter for assessment of respiratory function and potential technique errors are potential additional factors that influence a possibly more important improvement (30, 31).

In the post-hoc analysis regarding treatment response by age of onset, the VLOMG subgroup showed a lower CMI rate compared to both the EOMG and LOMG participants in our cohort, but also compared to the previously described VLOMG cohorts (86.7–97.6%) (32, 33); taking into consideration the overall data, the discrepancy is likely due to the small sample size in our VLOMG subgroup rather than a true differential effect. Regarding thymus status, while there were no differences between thymectomized and non-thymectomized patients or when taking into consideration histology subtypes, the limited number of patients in each subgroup precludes a conclusion.

Corticosteroid dose reduction was achieved for most participants, and three patients discontinued this therapy. Literature data support an overall corticosteroid-sparing effect for efgartigimod, potentially cumulative over time (32), but the degrees vary, mainly because of the different follow-up periods and different methods of reporting the changes (i.e., means versus medians of dose reduction) (22, 26, 34, 35).

Regarding treatment safety, three patients stopped therapy due to reasons perceived in relation to efgartigimod (one recurrent urinary tract infections, two with myasthenic crises and perceived lack of efficacy). Non-response or limited-response to efgartigimod have been previously reported (26, 27, 34). The two patients from our cohort presented myasthenic crises early in their efgartigimod therapy (after the first and second cycle, respectively) but had treatment response up to that point, and the drug was discontinued as per treating neurologists’ decision; the limited number of cases precludes a conclusion as to whether these events were due to reduced drug efficacy, and we believe that they were more likely due to the natural course of the disease.

Infections, specifically respiratory and urinary tract, are some of the most reported AEs in persons treated with efgartigimod, both in cohorts (18, 23, 27, 34) and pharmacovigilance studies (37–39). In our cohort, only one patient had to stop treatment due to recurrent UTIs, while respiratory tract infections were mild but nevertheless led to cycle interruption.

There are several limitations to this study. First, while there was a set or criteria for retreatment it was ultimately left at the decision of the treating physician whether to administer another cycle of therapy even when the patient did not meet the MG-ADL or QMG threshold; we do not believe this impacts our findings in a negative way as, at worst, it reduces the overall potential magnitude of improvement. Second, the group was heterogeneous in terms of NCSIS therapy; however, background medication, both AChEIs and NCSIS doses remained stable in the majority of participants and as MG-ADL improvement did not differ between patients with or without changes, we believe that the observed response was not significantly confounded by concomitant medication adjustments. Third, as a single-arm observational study without a control group, the observed improvement cannot be attributed to efgartigimod alone, as natural disease fluctuation could have contributed and was probably responsible for a number of instances where patients did not receive additional treatment cycles.

While investigators from Eastern Europe have participated in the ADAPT trial and its extension, to the best of our knowledge this is the first real-world evidence report on the use of efgartigimod in AChR-positive MG from the region.

## Conclusion

This prospective multicenter study confirms the effectiveness of efgartigimod for rapid symptom improvement and corticosteroid sparing and its results are consistent with previously published data. As one of the largest real-world experience cohorts and the first from Eastern Europe, this study extends the existing body of evidence to a novel population and clinical setting.

## Data Availability

The datasets generated and/or analyzed during the current study are not publicly available due to patient privacy and institutional restrictions, but are available from the corresponding author on reasonable request.

## References

[1] Hehir MK Silvestri NJ. (2018) Generalized Myasthenia Gravis. Neurol Clin. 36:253–260. doi: 10.1016/j.ncl.2018.01.002, 29655448

[2] Gilhus NE Verschuuren JJ. Myasthenia gravis: subgroup classification and therapeutic strategies. Lancet Neurol. (2015) 14:1023–36. doi: 10.1016/S1474-4422(15)00145-3, 26376969

[3] Gilhus NE Tzartos S Evoli A . Myasthenia gravis. Nat Rev Dis Primers. (2019) 5:30. doi: 10.1038/s41572-019-0079-y 31048702

[4] Berrih-Aknin S Frenkian-Cuvelier M Eymard B. Diagnostic and clinical classification of autoimmune myasthenia gravis. J Autoimmun. (2014) 48-49:143–8. doi: 10.1016/j.jaut.2014.01.003, 24530233

[5] Mantegazza R Bernasconi P Cavalcante P. Myasthenia gravis: from autoantibodies to therapy. Curr Opin Neurol. (2018) 31:517–25. doi: 10.1097/WCO.0000000000000596, 30156572

[6] Ruff RL Lisak RP. Nature and action of antibodies in myasthenia gravis. Neurol Clin. (2018) 36:275–91. doi: 10.1016/j.ncl.2018.01.001, 29655450

[7] Tüzün E Christadoss P. Complement associated pathogenic mechanisms in myasthenia gravis. Autoimmun Rev. (2013) 12:904–11. doi: 10.1016/j.autrev.2013.03.003, 23537510

[8] Gilhus NE Skeie GO Romi F Lazaridis K Zisimopoulou P Tzartos S. Myasthenia gravis — autoantibody characteristics and their implications for therapy. Nat Rev Neurol. (2016) 12:259–68. doi: 10.1038/nrneurol.2016.44, 27103470

[9] Wiendl H Abicht A Chan A Della Marina A Hagenacker T Hekmat K . Guideline for the management of myasthenic syndromes. Ther Adv Neurol Disord. (2023) 16:17562864231213240. doi: 10.1177/17562864231213240, 38152089 PMC10752078

[10] De Bleecker JL Remiche G Alonso-Jiménez A . Recommendations for the management of myasthenia gravis in Belgium. Acta Neurol Belg. (2024) 124:1371–83. doi: 10.1007/s13760-024-02552-7, 38649556 PMC11266451

[11] Sanders DB Wolfe GI Benatar M Evoli A Gilhus NE Illa I . International consensus guidance for management of myasthenia gravis: executive summary. Neurology. (2016) 87:419–25. doi: 10.1212/WNL.0000000000002790, 27358333 PMC4977114

[12] Morren J Li Y. Maintenance immunosuppression in myasthenia gravis, an update. J Neurol Sci. (2020) 410:116648. doi: 10.1016/j.jns.2019.116648, 31901719

[13] Garcia-Garcia J Díaz-Maroto I Martínez-Martín A Pardal-Fernández JM Segura T. A series of patients with refractory myasthenia gravis. Neurologia. (2023) 38:256–61. doi: 10.1016/j.nrleng.2023.04.001, 37031801

[14] Habib AA. Refractory myasthenia gravis: the more we learn, the less we know. RRNMF Neuromuscul J. (2023) 4, 90–97. doi: 10.17161/rrnmf.v4i3.19555

[15] Mansour GK Alangari L Khosyfan L Alhammad R Hajjar AW. Efgartigimod for generalized myasthenia gravis and beyond: a narrative review of its pharmacological profile, clinical utility, and expanding applications. Biomedicine. (2025) 13:2975. doi: 10.3390/biomedicines13122975, 41462987 PMC12730960

[16] Gable KL Guptill JT. Antagonism of the neonatal fc receptor as an emerging treatment for myasthenia gravis. Front Immunol. (2020) 10:3052. doi: 10.3389/fimmu.2019.03052, 31998320 PMC6965493

[17] Saccà F Barnett C Vu T Peric S Phillips GA Zhao S . Efgartigimod improved health-related quality of life in generalized myasthenia gravis: results from a randomized, double-blind, placebo-controlled, phase 3 study (ADAPT). J Neurol. (2023) 270:2096–105. doi: 10.1007/s00415-022-11517-w, 36598575 PMC10025199

[18] Howard JF Bril V Vu T . Safety, efficacy, and tolerability of efgartigimod in patients with generalised myasthenia gravis (ADAPT): a multicentre, randomised, placebo-controlled, phase 3 trial. Lancet Neurol. (2021) 20:526–36. doi: 10.1016/S1474-4422(21)00159-9, 34146511

[19] Mendoza M Tran C Bril V Katzberg HD Barnett C. Patient-acceptable symptom states in myasthenia gravis. Neurology. (2020) 95:e1617–28. doi: 10.1212/WNL.0000000000010574, 32759200

[20] Spagni G Verza MU Cornacchini S Beretta F Sun B Lotti A . Validation of the “patient-acceptable symptom state” question as outcome measure in AChR myasthenia gravis: a multicentre. Prospective Study Eur J Neurol. (2025) 32:e70262. doi: 10.1111/ene.70262, 40556475 PMC12188101

[21] Bril V Howard JF Karam C . Effect of efgartigimod on muscle group subdomains in participants with generalized myasthenia gravis: post hoc analyses of the phase 3 pivotal ADAPT study. Eur J Neurol. (2024) 31:e16098. doi: 10.1111/ene.16098, 37843174 PMC11235734

[22] Fuchs L Shelly S Vigiser I Kolb H Regev K Schwartzmann Y . Real-world experience with efgartigimod in patients with myasthenia gravis. J Neurol. (2024) 271:3462–70. doi: 10.1007/s00415-024-12293-5, 38528163

[23] Katyal N Halldorsdottir K Govindarajan R Shieh P Muley S Reyes P . Safety and outcomes with efgartigimod use for acetylcholine receptor-positive generalized myasthenia gravis in clinical practice. Muscle Nerve. (2023) 68:762–6. doi: 10.1002/mus.27974, 37695277

[24] Hao S Ruan Z Guo R Wang Q Huang X Sun C . Efficacy and safety of Efgartigimod for patients with myasthenia gravis in a real-world cohort of 77 patients. CNS Neurosci Ther. (2025) 31:e70391. doi: 10.1111/cns.70391, 40237260 PMC12001068

[25] Niu Z Wang J Ren J Liu R Guo J Zhang N . Real-world experience with efgartigimod in generalized myasthenia gravis: a single-center retrospective study in China. Front Immunol. (2026) 17:1752846. doi: 10.3389/fimmu.2026.1752846, 42125648 PMC13158079

[26] Remijn-Nelissen L Tannemaat MR Ruiter AM Campman YJM Verschuuren JJGM. Efgartigimod in refractory autoimmune myasthenia gravis. Muscle Nerve. (2024) 70:325–32. doi: 10.1002/mus.28184, 38899431

[27] Moniz Dionísio J Ambrose P Burke G Farrugia ME Garcia-Reitboeck P Hewamadduma C . Efgartigimod efficacy and safety in refractory myasthenia gravis: UK’S first real-world experience. J Neurol Neurosurg Psychiatry. (2025) 96:322–8. doi: 10.1136/jnnp-2024-334086, 39798959

[28] Sgarzi M Paone P Camera G Agazzi E Mazzoleni S Martorana F . Real world study in Italian public hospital with Efgartigimod in patients affected by generalized myasthenia gravis: influence of clinical and serological factors. Front Neurol. (2025) 16:1555068. doi: 10.3389/fneur.2025.1555068, 40401017 PMC12092212

[29] Pane C Di Stefano V Cuomo N . A real-life experience with eculizumab and efgartigimod in generalized myasthenia gravis patients. J Neurol. (2024) 271:6209–19. doi: 10.1007/s00415-024-12588-7, 39080054 PMC11377599

[30] Sakhamuri S Seemungal T. Beyond airflow obstruction: acknowledging the diversity of abnormal spirometry patterns. ERJ Open Res. (2023) 9:00193–2023. doi: 10.1183/23120541.00193-2023, 37404846 PMC10316041

[31] Varelas PN Lopez-Plaza I Ata A Rehman MF Mehta C Ramadan R . Longitudinal improvement in respiratory function following plasma exchange in patients with severe myasthenia gravis. Neurocrit Care. (2025) 43:458–66. doi: 10.1007/s12028-025-02238-9, 40180670

[32] Ma C Shen J Zhu Y Wang B Zhu R. Efgartigimod in very-late-onset generalized myasthenia gravis: a real-world study on effectiveness and safety. Curr Neuropharmacol. (2026) 24:859–66. doi: 10.2174/011570159X415163251205123550, 41572778 PMC13519559

[33] Zhang Z Yang M Guo X Ma T Wang Z Luo T . Efgartigimod as a fast-acting treatment in generalized very-late-onset myasthenia gravis. Front Immunol. (2025) 16:1579859. doi: 10.3389/fimmu.2025.1579859, 40313945 PMC12043626

[34] Singer M Khella S Bird S McIntosh P Paudyal B Wadhwani A . Single institution experience with efgartigimod in patients with myasthenia gravis: patient selection, dosing schedules, treatment response, and adverse events. Muscle Nerve. (2024) 69:87–92. doi: 10.1002/mus.28003, 37990374

[35] Nomura T Imamura M Imura M Mizutani H Ueda M. Efgartigimod treatment for generalized myasthenia gravis: a single-center case series of 16 patients. Front Neurol. (2024) 15:1472845. doi: 10.3389/fneur.2024.1472845, 39469071 PMC11514137

[36] Luo S Jiang Q Zeng W Wang Q Zou Z Yu Y . Efgartigimod for generalized myasthenia gravis: a multicenter real-world cohort study in China. Ann Clin Transl Neurol. (2024) 11:2212–21. doi: 10.1002/acn3.52142, 38973109 PMC11330228

[37] Huang J Ye H Lin J Luo D Huang P Zheng X. Post-marketing safety concerns with Efgartigimod alfa: a pharmacovigilance analysis based on the Food and Drug Administration adverse event reporting system database. CLEP Epidemiol. (2025) 17:765–78. doi: 10.2147/CLEP.S514738, 40977954 PMC12449886

[38] Yang Y Liu J Wei W. A real-world pharmacovigilance study of efgartigimod alfa in the FDA adverse event reporting system database. Front Pharmacol. (2025) 16:1510992. doi: 10.3389/fphar.2025.1510992, 40308753 PMC12041031

[39] Ju Y Qin X. Postmarketing adverse events of efgartigimod alfa: a real-world pharmacovigilance study based on the FAERS database. BMJ Open. (2025) 15:e095652. doi: 10.1136/bmjopen-2024-095652, 40659410 PMC12258281

