# Supplementary material for: Efgartigimod-alfa therapy in acetylcholine receptor antibody myasthenia gravis: Romanian experience
Source: Front Neurol. 2026 Sep 3;17:1945691. doi: 10.3389/fneur.2026.1945691 (PMC13583037; doi:10.3389/fneur.2026.1945691)
Supplement: Supplementary file 1 [file Table_1.docx]

| MG-ADL reduction | | QMG reduction | |
| --- | --- | --- | --- |
| ≥2 points* | 89.7% | ≥3 points* | 74.4% |
| ≥3 points† | 76.9% | ≥5 points† | 56.4% |
| ≥5 points | 46.2% | ≥7 points | 41.0% |
| ≥7 points | 25.6% | ≥9 points | 30.8% |

**Supplementary table 1. Magnitude of MG-ADL and QMG response during the first cycle of treatment**

*degrees of reduction selected as per protocol thresholds; †degrees of reduction selected as per ADAPT post-hoc analysis (Dewilde S et al. J Neurol Sci. 2024 Nov 15;466:123264. doi: 0.1016/j.jns.2024.123264); other thresholds are exploratory
