# Supplementary material for: Efgartigimod-alfa therapy in acetylcholine receptor antibody myasthenia gravis: Romanian experience
Source: Front Neurol. 2026 Sep 3;17:1945691. doi: 10.3389/fneur.2026.1945691 (PMC13583037; doi:10.3389/fneur.2026.1945691)
Supplement: Supplementary file 2 [file Table_2.docx]

**Supplementary table 2.1. Subdomain analysis for MG-ADL scores for treatment cycles 1 - 8**

| **Cycle no.**  **(participants)** | **Bulbar** | | | | **Respiratory** | | | | **Limb** | | | | **Ocular** | | | |
| --- | --- | --- | --- | --- | --- | --- | --- | --- | --- | --- | --- | --- | --- | --- | --- | --- |
|  | **1^st^ infusion** | **4^th^ infusion** | **Change** | **p-value** | **1^st^ infusion** | **4^th^ infusion** | **Change** | **p-value** | **1^st^ infusion** | **4^th^ infusion** | **Change** | **p-value** | **1^st^ infusion** | **4^th^ infusion** | **Change** | **p-value** |
| Cycle 1  (n = 39) | 3.0 | 0.0 | 2.0 | <0.001 | 1.0 | 0.0 | 0.0 | <0.001 | 2.0 | 0.0 | 1.0 | <0.001 | 1.0 | 0.0 | 1.0 | <0.001 |
| Cycle 2  (n = 37) | 2.0 | 0.0 | 1.0 | <0.001 | 1.0 | 0.0 | 0.0 | 0.0317 | 1.0 | 0.0 | 1.0 | <0.001 | 1.0 | 0.0 | 1.0 | <0.001 |
| Cycle 3  (n = 33) | 3.0 | 0.0 | 2.0 | <0.001 | 1.0 | 0.0 | 0.0 | 0.0052 | 1.0 | 0.0 | 1.0 | <0.001 | 1.0 | 0.0 | 1.0 | 0.0012 |
| Cycle 4  (n = 30) | 3.0 | 0.0 | 2.0 | <0.001 | 1.0 | 0.0 | 0.0 | 0.0018 | 1.0 | 0.5 | 0.0 | 0.1106 | 1.0 | 0.0 | 1.0 | <0.001 |
| Cycle 5  (n = 23) | 3.0 | 0.0 | 2.0 | <0.001 | 1.0 | 0.0 | 0.0 | 0.0103 | 2.0 | 0.0 | 1.0 | 0.0073 | 2.0 | 1.0 | 0.0 | 0.0035 |
| Cycle 6  (n = 17) | 3.0 | 0.0 | 2.0 | <0.001 | 1.0 | 0.0 | 0.0 | 0.0726 | 2.0 | 0.0 | 1.0 | 0.0090 | 1.0 | 0.0 | 1.0 | 0.0022 |
| Cycle 7  (n = 12) | 2.5 | 0.0 | 2.5 | 0.0023 | 1.0 | 0.0 | 0.5 | 0.0263 | 2.0 | 0.0 | 1.0 | 0.0042 | 2.0 | 0.0 | 1.0 | 0.0206 |
| Cycle 8  (n = 11) | 3.0 | 1.0 | 2.0 | 0.0048 | 1.0 | 0.0 | 1.0 | 0.0083 | 2.0 | 0.0 | 2.0 | 0.0086 | 2.0 | 0.0 | 2.0 | 0.0048 |

Values represent medians. Change = median of individual paired differences (1st infusion − 4th infusion). Wilcoxon signed-rank test. MG-ADL subdomains: Bulbar (items 1–3: talking, chewing, swallowing; range 0–9), Respiratory (item 4: breathing; range 0–3), Limb (items 5–6: brushing teeth/combing hair, arising from chair; range 0–6), Ocular (items 7–8: double vision, eyelid droop; range 0–6).

**Supplementary table 2.2. Subdomain analysis for QMG scores for treatment cycles 1 - 8**

| **Cycle no.**  **(participants)** | **Ocular** | | | | **Bulbar** | | | | **Limb** | | | | **Respiratory** | | | | **Axial** | | | |
| --- | --- | --- | --- | --- | --- | --- | --- | --- | --- | --- | --- | --- | --- | --- | --- | --- | --- | --- | --- | --- |
|  | **1^st^ infusion** | **4^th^ infusion** | **Change** | **p-value** | **1^st^ infusion** | **4^th^ infusion** | **Change** | **p-value** | **1^st^ infusion** | **4^th^ infusion** | **Change** | **p-value** | **1^st^ infusion** | **4^th^ infusion** | **Change** | **p-value** | **1^st^ infusion** | **4^th^ infusion** | **Change** | **p-value** |
| Cycle 1  (n = 39) | 3.0 | 1.0 | 1.0 | <0.001 | 1.0 | 0.0 | 1.0 | <0.001 | 8.5 | 5.0 | 3.0 | <0.001 | 0.0 | 0.0 | 0.0 | 0.0953 | 1.0 | 1.0 | 0.0 | <0.001 |
| Cycle 2  (n = 37) | 2.0 | 1.0 | 1.0 | 0.0041 | 1.0 | 0.0 | 0.0 | <0.001 | 7.0 | 4.5 | 2.5 | <0.001 | 0.0 | 0.0 | 0.0 | 0.2330 | 1.0 | 1.0 | 0.0 | <0.001 |
| Cycle 3  (n = 33) | 2.0 | 1.0 | 1.0 | <0.001 | 1.0 | 0.0 | 1.0 | <0.001 | 7.0 | 5.0 | 2.0 | <0.001 | 0.0 | 0.0 | 0.0 | 0.5297 | 1.0 | 1.0 | 1.0 | <0.001 |
| Cycle 4  (n = 30) | 2.0 | 0.0 | 2.0 | <0.001 | 1.0 | 0.0 | 0.0 | 0.0014 | 8.0 | 5.0 | 2.0 | <0.001 | 0.0 | 0.0 | 0.0 | 0.0147 | 1.0 | 0.5 | 1.0 | <0.001 |
| Cycle 5  (n = 23) | 2.0 | 1.0 | 2.0 | <0.001 | 1.0 | 0.0 | 0.0 | 0.0086 | 8.0 | 5.0 | 2.0 | 0.0010 | 0.0 | 0.0 | 0.0 | 0.2330 | 1.0 | 1.0 | 1.0 | <0.001 |
| Cycle 6  (n = 17) | 3.0 | 1.0 | 1.0 | 0.0022 | 1.0 | 0.0 | 1.0 | 0.0079 | 8.0 | 6.0 | 1.0 | 0.0502 | 0.0 | 0.0 | 0.0 | 0.0719 | 1.0 | 1.0 | 0.0 | 0.0197 |
| Cycle 7  (n = 12) | 2.5 | 0.0 | 2.0 | 0.0035 | 1.0 | 0.0 | 1.0 | 0.0401 | 8.0 | 4.5 | 3.0 | 0.0036 | 0.0 | 0.0 | 0.0 | NA | 1.5 | 1.0 | 1.0 | 0.0107 |
| Cycle 8  (n = 11) | 2.0 | 0.0 | 2.0 | 0.0086 | 0.0 | 0.0 | 0.0 | 0.0545 | 8.0 | 5.0 | 3.0 | 0.0037 | 0.0 | 0.0 | 0.0 | 0.3458 | 2.0 | 1.0 | 1.0 | 0.0069 |

Values represent medians. Change = median of individual paired differences (1st infusion − 4th infusion). Wilcoxon signed-rank test. QMG subdomains: Ocular (items 1–3: double vision, ptosis, facial muscles; range 0–9), Bulbar (items 4–5: swallowing, speech; range 0–6), Limb (items 6–7, 9–10, 12–13: arms outstretched, hand grip, legs outstretched; range 0–18), Respiratory (item 8: vital capacity; range 0–3), Axial (item 11: head lift; range 0–3). The respiratory subdomain did not reach statistical significance in any cycle. NA = test not computable (all paired differences were zero).
