# Supplementary material for: Efgartigimod-alfa therapy in acetylcholine receptor antibody myasthenia gravis: Romanian experience
Source: Front Neurol. 2026 Sep 3;17:1945691. doi: 10.3389/fneur.2026.1945691 (PMC13583037; doi:10.3389/fneur.2026.1945691)
Supplement: Supplementary file 3 [file Table_3.docx]

**Supplementary table 3. Exploratory subgroups analysis of treatment response by age of MG onset and thymus histology for patients included in the efficacy analysis (n = 39).**

| **Subgroup** | **n** | **CMI cycle 1**  **n (%)** | **MG-ADL change**  **(median)** | **Number of cycles**  **(median [Q1-Q3])** | **p** |
| --- | --- | --- | --- | --- | --- |
| **By age of onset** | | | | | |
| EOMG | 24 | 23 (95.8%) | 4.5 | 6 [4-8] |  |
| LOMG | 9 | 9 (100%) | 4.0 | 5 [4-5] |  |
| VLOMG | 6 | 3 (50.0%) | 1.3 | 4 [2-5] |  |
| CMI across groups |  |  |  |  | 0.002* |
| MG-ADL change across groups |  |  |  |  | 0.359† |
| **By thymectomy status** | | | | | |
| Prior thymectomy | 19 | 18 (94.7%) | 4.0 | 4 [4-7] |  |
| No thymectomy | 20 | 17 (85.0%) | 4.0 | 5 [4-6] |  |
| CMI |  |  |  |  | 0.605‡ |
| MG-ADL change |  |  |  |  | 0.372§ |
| **By thymus histology** | | | | | |
| Thymoma | 5 | 5 (100%) | 6.0 | 5 [2-8] |  |
| Hyperplasia | 6 | 6 (100%) | 4.0 | 7 [4-8] |  |
| Normal | 2 | 1 (50%) | 2.5 | 4 [4-4] |  |
| Unknown | 6 | 6 (100%) | 5.0 | 4 [4-7] |  |
| MG-ADL change (known histology) | 13 |  |  |  | 0.262† |

CMI - clinically meaningful improvement (≥2-point MG-ADL reduction); MG-ADL change - median reduction from the first to the last visit of each cycle

*Chi-square test; † Kruskal-Wallis test; ‡Fisher’s exact test; §Mann-Whitney U test
