# Supplementary material for: Efgartigimod-alfa therapy in acetylcholine receptor antibody myasthenia gravis: Romanian experience
Source: Front Neurol. 2026 Sep 3;17:1945691. doi: 10.3389/fneur.2026.1945691 (PMC13583037; doi:10.3389/fneur.2026.1945691)
Supplement: Supplementary file 4 [file Table_4.docx]

**Supplementary table 3. Background, non-corticosteroid, medication changes during the study period (for patients included in the efficacy analysis, n = 39).**

|  | **AChEI**  **(n = 39)** | **Azathioprine**  **(n = 13*)** | **Mycophenolate mofetil**  **(n = 5*)** |
| --- | --- | --- | --- |
| On medication at enrollment, n (%)† | 39 (100%) | 11 (28.2%) | 3 (7.7%) |
| Started during follow-up period, n (%)† | 0 | 2 (5.1%) | 2 (5.1%) |
| Baseline dose, median [Q1-Q3] | 240 [180-300] mg | 100 [100-100] mg | 1500 [1500-1750] mg |
| Final dose, median [Q1-Q3] | 240 [180-255] mg | 100 [100-100] mg | 2000 [1500-2000] mg |
| Dose unchanged, n (%)‡ | 29 (74.4%) | 9 (69.2%) | 2 (40.0%) |
| Dose decreased, n (%)‡ | 8 (20.5%) | 0 | 0 |
| Dose increased, n (%)‡ | 2 (5.1%) | 0 | 1 (20.0%) |
| Discontinued, n (%)‡ | 0 | 2 (15.4%) | 0 |
| Wilcoxon signed-rank p | 0.026 | 0.500 | 1.000 |

*total number of patients receiving this medication at any point during the study

†percentages are calculated from the total number of patients included in the efficacy analysis

‡percentages are calculated from the total number of patients receiving the medication at any point during the study
