# Supplementary material for: Efgartigimod-alfa therapy in acetylcholine receptor antibody myasthenia gravis: Romanian experience
Source: Front Neurol. 2026 Sep 3;17:1945691. doi: 10.3389/fneur.2026.1945691 (PMC13583037; doi:10.3389/fneur.2026.1945691)
Supplement: Supplementary file 5 [file Table_5.docx]

**Supplementary table 3.1. Baseline characteristics and cycle 1 CMI rates across efgartigimod studies**

| **Study** | **N** | **Age (years)** | **Gender (female, %)** | **MGFA III+ (%)** | **MG-ADL (baseline)** | **CMI (%)** | **Assessment** |
| --- | --- | --- | --- | --- | --- | --- | --- |
| present study | 39* | 51.1±17.0 | 61.0 | 41.5 | 7.9±2.3 | 89.7 | end-of-cycle |
| Howard, 2021 (ADAPT AChR+) | 65 | 44.7±15.0 | 70.8 | 56.9 | 9.0±2.5 | 67.7 | sustained ≥4 weeks |
| Katyal, 2023 | 36* | 65.6±14.7 | 37.8 | 75.7 | 8.0±3.1 | 72.2 | end-of-cycle |
| Fuchs, 2024 | 22 | 58.1±20.2 | 50.0 | 72.7 | 6.5 (3-17)† | 90.9 | 4 weeks |
| Remijn-Nelissen, 2024 | 16 | 47.2±16.8 | 68.8 | ―‡ | 7.9±4.5 | 62.5 | sustained ≥4 weeks |
| Dionísio, 2025 | 48 | 49.2±14.2 | 75.0 | ―‡ | 11.2±3.2 | 75.0 | sustained ≥4 weeks |
| Hao, 2025 | 76* | 56.1±15.2 | 40.3 | 50.6 | 7.7±3.6 | 98.7 | 4 weeks |
| Niu, 2026 | 81 | 67.0 (55-76)† | 69.1 | 32.1 | 8.08±4.28 | 80.3 | 4 weeks |

*Efficacy cohort. †Reported as median (Q1, Q3). ‡Specific MGFA distribution not stated.

CMI = clinically meaningful improvement (≥2 point MG-ADL reduction) after cycle 1

**Supplementary table 3.2. Spearman rank correlations between study-level baseline characteristics and cycle 1 CMI rates**

| **Variable** | **Number of studies** | **Spearman r** | **p-value** |
| --- | --- | --- | --- |
| Age (mean) | 8 | 0.500 | 0.207 |
| Gender (female, %) | 8 | -0.405 | 0.320 |
| MGFA III+ (%) | 6 | -0.200 | 0.704 |
| MG-ADL (baseline, mean) | 7 | -0.378 | 0.403 |
| Thymectomy (%) | 7 | -0.464 | 0.294 |
| Thymoma (%) | 5 | 0.200 | 0.747 |

Note: study-level correlations.
